# Influence of meteorological factors on development of spontaneous pneumothorax

**DOI:** 10.1097/MD.0000000000031488

**Published:** 2022-11-11

**Authors:** Suk Hee Lee, Young Woo Seo, Sang Gyu Kwak

**Affiliations:** a Department of Emergency Medicine, College of Medicine, Daegu Catholic University, Daegu, Korea; b Department of Medical Statistics, College of Medicine, Daegu Catholic University, Daegu, Korea.

**Keywords:** atmospheric pressure, meteorological factors, spontaneous pneumothorax, temperature, wind speed

## Abstract

This study investigated the correlation between spontaneous pneumothorax (SP) and meteorological factors during different seasons. Patients who visited emergency rooms (ERs) in large cities in Korea and were discharged with SP from 2014 to 2016 were included in this study. Data on temperature, air pressure, and wind speed for each region were collected to obtain each factor’s daily maximum, minimum, average, and changes. Days with more than 1 case of SP per million were referred to as pneumothorax days (PD) and those with less than 1 case of SP per million were referred to as non-pneumothorax days (NPD). The environmental factors were assessed on the same day (Day 0), 1 day prior (Day-1), and 2 days prior (Day-2) to PD and NPD per season. A total of 17,846 patients were included in this study. During winter, 4080 patients with SP visited the ERs of large cities with low population densities. The maximum temperature (0.16°C vs 0.76°C, 0.04°C vs 0.87°C, and 0.09°C vs 0.91°C), change in temperature (0.24°C vs 0.90°C, 0.38°C vs 0.81°C, and 0.41°C vs 0.83°C), average atmospheric pressure (0.16 vs 0.52 hPa, 0.25 vs 0.42 hPa, 0.34 vs 0.40 hPa), and maximum atmospheric pressure (0.15 vs 0.53 hPa, 0.28 vs 0.49 hPa, 0.33 vs 0.71 hPa) were greater for Day 0, Day-1, and Day-2, respectively, in PD than in NPD. Meanwhile, the average (0.31 vs 0.48 m/s, 0.28 vs 0.46 m/s, 0.20 vs 0.40 m/s), minimum (0.20 vs 0.31 m/s, 0.18 vs 0.25 m/s, 0.16 vs 0.25 m/s), and maximum (0.44 vs 0.67 m/s, 0.36 vs 0.71 m/s, 0.26 vs 0.58 m/s) wind speeds were slower, and the changes in wind speed (0.44 vs 0.67 m/s, 0.36 vs 0.71 m/s, 0.16 vs 0.25 m/s) were lower for all 3 days in PD than in NPD. High average and change in temperature, slow and unchanging wind speed, and high average and maximum atmospheric pressure were associated with SP. Since many findings of this study were contradictory to previous studies, it is assumed that the interaction of various factors affects SP.

## 1. Introduction

Spontaneous pneumothorax (SP) is characterized by air in the pleural space caused by the rupture of the lung bullae and/or blebs without any trauma. In previous studies, changes in the airway pressure were suspected of causing bleb rupture,^[1–3]^ and some have already assessed the effects of climate and environmental factors, such as barometric pressure, changes in air pressure, and temperature variations on the development of pneumothorax.^[4–7]^ Despite these studies, the exact cause of pneumothorax is still unknown.^[8]^ Previous reports were focused on single-center studies of large cities, and there is a lack of reports using cross-country data collected by the national government. Additionally, the atmospheric and meteorological conditions differ in each season, and only a few studies have compared the effects of these conditions on SP. Therefore, this study aimed to investigate the correlation between SP and seasonal environmental factors using the data of patients in emergency centers provided by the Central Emergency Medical Center of Korea and weather information obtained from the Korea Meteorological Administration.

## 2. Methods

### 2.1. Study period and participants

Data of patients who visited emergency rooms (ERs) in Korea and were discharged or diagnosed with SP (ICD-10, The international statistical classification of diseases and related health problems, J93) from January 1, 2014, to December 31, 2016, were obtained from the National Emergency Department Information System (NEDIS) and were included in this study.

**Figure 1.**
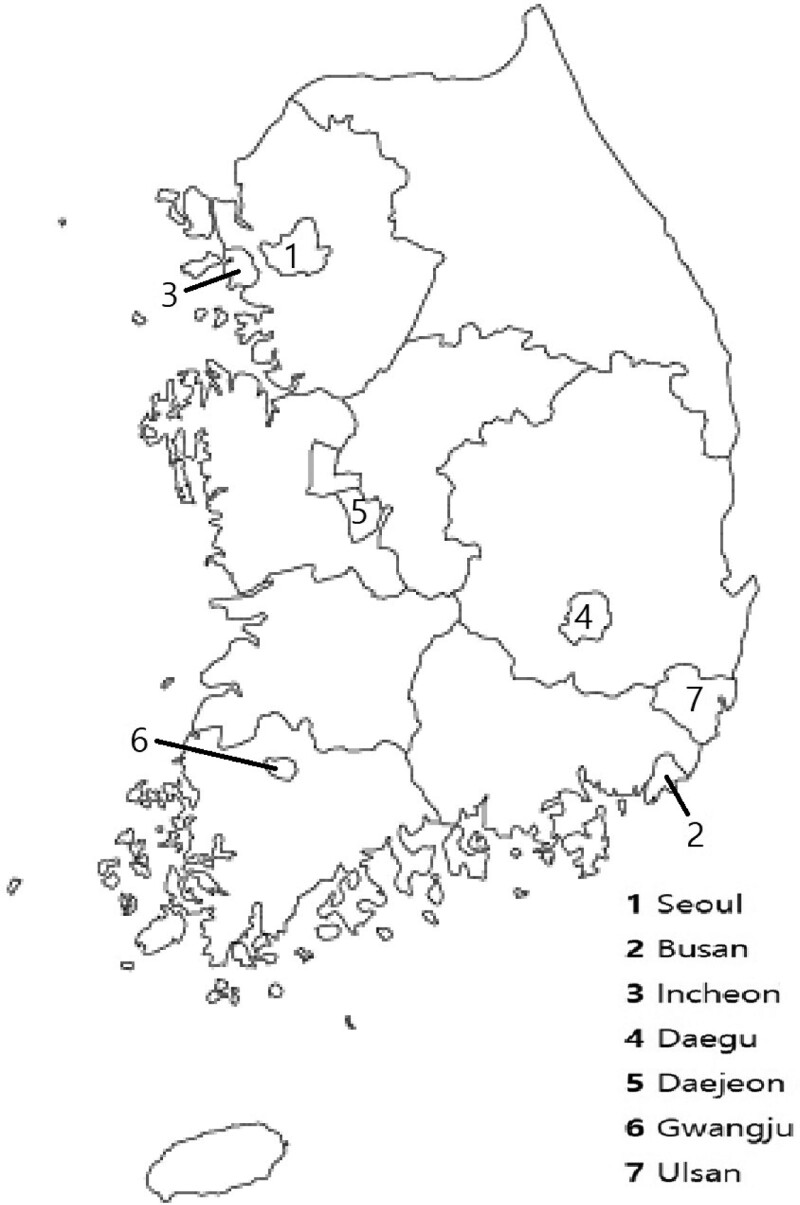
The map of Korea. The numbers denote the different metropolitan cities in Korea.

The NEDIS database provided detailed information on metropolitan and provincial regions among the administrative districts in Korea. Provincial regions include many rural, large areas with small populations and low population densities. The NEDIS data only provides the area code of the emergency medical center visited by the patient. As a result, there was a high risk of error when collecting patient data taken from provincial regions with widely distributed hospitals. Therefore, the data included in this study were from Seoul, Busan, Incheon, Daegu, Daejeon, Gwangju, and Ulsan, which are large and highly populated cities with small geographic areas (Fig. 1). Meanwhile, data from patients who visited hospitals in provincial regions, which have low population densities, were excluded.

Between 2014 and 2016, there were 7 metropolitan cities in Korea: Seoul, Busan, Incheon, Daegu, Daejeon, Gwangju, and Ulsan. Each of these cities had smaller areas than provincial regions, with a population of over 1 million and high population densities.

* NEDIS has been collecting data from emergency medical institutions in Korea since June 2003. Emergency medical institutions computerize the patient’s name, date of birth, date and time of the disease onset, and diagnostic codes, which are sent to the Central Emergency Medical Center. This data is collected and managed by the National Emergency Medical Center to evaluate the quality of emergency medical institutions in Korea and prepare annual statistical reports.

### 2.2. Study methods

A total of 21,273 patients with SP visited emergency medical institutions located in the 7 metropolitan cities during the study period. Among them, 3427 patients were excluded for insufficient information, such as missing dates and onset of disease data. The data of 17,846 patients were included in the final analysis. The data on the population and area by region were obtained from the database of Statistics Korea and the Ministry of Public Administration and Security. The days with more than 1 patient with SP per 1 million population were referred to as pneumothorax days (PD). The days with less than 1 patient with SP per 1 million population were referred to as non-pneumothorax days (NPD).

The weather data was obtained from the Korea Meteorological Administration. Each of the large cities had 1 or 2 weather stations. Data from the station closest to the city center was selected for the cities with 2 weather stations. Temperature (°C), wind speed (m/s), and atmospheric pressure (hPa) were collected for each region and time. The daily maximum, minimum, and change (change = maximum - minimum) per day were calculated for each factor. The data on each factor for PD and NPD were taken on the day of (Day 0), 1 day before (Day-1), and 2 days before (Day-2) and were compared per region.

### 2.3. Statistical analysis

SPSS version 21.0 for Windows (SPSS Inc., Chicago, IL) was used for all statistical analysis. Shapiro–Wilk test was conducted to assess the normality of continuous variables. Student *t* test or Mann–Whitney *U* test was used according to the normal distribution of variables. Analysis of covariance (ANCOVA) was conducted to correct for differences by season. A *P* value of < .05 was considered statistically significant.

### 2.4. Research ethics

This study was approved by the Institutional Review Board (IRB) of Daegu Catholic University Hospital (IRB number: CR-18-049-L). Informed consent was not required.

## 3. Results

### 3.1. Demographic and clinical characteristics of patients with SP

There are 7 metropolises in Korea, with a total population of about 23 million and an area of 5407.77 km^2^. Seoul is the capital of Korea and has a population of 9.95 million and an area of 605.23km^2^. Busan is a major port city located in southeast Korea and has a population and area of 3.47 million and 769.83 km^2^, respectively. Incheon, the port city of the Yellow Sea, is in the western part of Seoul and has a population of 2.89 million and an area of 1047.74 km^2^. Daegu is the representative city of the inland region in southeast Korea and has a population of 2.48 million and an area of 883.56 km^2^. Daejeon is located in the central inland part, with a population of 1.54 million and an area of 539.32 km^2^. Gwangju is the representative city of the southwest, with a population of 1.49 million and an area of 501.17 km^2^. Ulsan is an industrial coastal city in the southeast, with a population of 1.16 million and an area of 1060.86 km^2^ (Table 1).

**Table 1:** Demographic and clinical characteristics of patients with spontaneous pneumothorax who visited hospitals within the metropolis.

Metropolis	Characteristics of metropolis		Characteristics of patients			Season			
	Mean population (million)	Area (km^2^)	Patients (N)	Age (Mean ± SD)	Male N (%)	Spring N (%)	Summer N (%)	Autumn N (%)	Winter N (%)
All cities	22.99	5407.70	17,846	37.32 ± 22.77	15,451 (86.58)	4671 (26.17)	4517 (25.31)	4578 (25.65)	4080 (22.86)
Seoul	9.95	605.23	9090	36.43 ± 22.10	7770 (85.48)	2428 (26.71)	2239 (24.63)	2331 (25.64)	2092 (23.01)
Busan	3.47	769.83	1674	40.22 ± 23.68	1461 (87.28)	480 (28.67)	413 (24.67)	373 (22.28)	408 (24.37)
Incheon	2.89	1047.74	1591	36.81 ± 22.40	1390 (87.37)	375 (23.57)	389 (24.45)	465 (29.23)	362 (22.75)
Daegu	2.48	883.56	2435	38.90 ± 23.66	2138 (87.80)	645 (26.49)	619 (25.42)	622 (25.54)	549 (22.55)
Daejeon	1.54	539.32	1779	32.55 ± 22.87	1557 (87.52)	435 (24.45)	485 (27.26)	461 (25.91)	398 (22.37)
Gwangju	1.49	501.17	751	41.24 ± 24.60	664 (88.42)	171 (22.77)	222 (29.56)	196 (26.10)	162 (21.57)
Ulsan	1.16	1060.86	526	34.06 ± 21.99	471 (89.54)	137 (26.05)	150 (28.52)	130 (24.71)	109 (20.72)

The mean age of patients with SP was 37.32 ± 22.77 years, and 15,451 patients (86.58%) were male. By season, 4671 (26.17%), 4517 (25.31%), 4578 (25.65%), and 4080 (22.86%) patients were reported in spring, summer, autumn, and winter, respectively. The incidence rate of SP was observed to be lower in the winter than in other seasons (Table 1).

### 3.2. Comparisons of temperature

There were no significant differences in the average and minimum temperatures between PD and NPD. In the 3-day comparison, the maximum temperature was greater by 0.16°C versus 0.76°C on Day 0, 0.04°C versus 0.87°C on Day-1, and 0.09°C versus 0.91°C on Day-2 in PD than in NPD. Additionally, the temperature change was greater by 0.24°C versus 0.90°C on Day 0, 0.38°C versus 0.81°C on Day-1, and 0.41°C versus 0.83°C on Day-2 in PD than in NPD (Table 2).

**Table 2:** Comparison of temperature between pneumothorax days and non-pneumothorax days adjusted by seasonal variations.

	Day	Group	Season				*P*
			Spring °C (±SD)	Summer °C (±SD)	Autumn °C (±SD)	Winter °C (±SD)	
Mean	0	NPD	13.74 (5.62)	24.57 (2.77)	16.18 (5.83)	2.32 (4.31)	.063
		PD	13.98 (5.90)	25.00 (2.85)	16.27 (5.84)	2.41 (3.80)	
	−1	NPD	13.54 (5.67)	24.54 (2.77)	16.33 (5.80)	2.44 (4.30)	.109
		PD	13.74 (5.91)	25.03 (2.85)	16.45 (5.85)	2.36 (3.92)	
	−2	NPD	13.37 (5.70)	24.53 (2.80)	16.52 (5.76)	2.46 (4.28)	.107
		PD	13.54 (5.88)	25.03 (2.85)	16.59 (5.79)	2.43 (3.98)	
Min	0	NPD	10.70 (4.69)	21.56 (2.81)	13.46 (5.23)	1.30 (4.07)	.170
		PD	10.40 (5.11)	21.58 (2.78)	13.38 (5.14)	1.10 (3.76)	
	−1	NPD	10.55 (4.76)	21.52 (2.82)	13.62 (5.17)	1.37 (4.10)	.105
		PD	10.21 (5.06)	21.58 (2.82)	13.47 (5.21)	1.14 (3.86)	
	−2	NPD	10.40 (4.85)	21.49 (2.84)	13.80 (5.12)	1.38 (4.07)	.096
		PD	10.08 (4.96)	21.58 (2.85)	13.56 (5.18)	1.19 (3.95)	
Max	0	NPD	15.98 (7.58)	28.31 (3.39)	18.92 (7.39)	5.35 (4.89)	.002
		PD	16.14 (8.00)	29.07 (3.53)	19.08 (7.65)	6.05 (4.18)	
	−1	NPD	15.78 (7.57)	28.29 (3.39)	19.08 (7.35)	5.45 (4.88)	.004
		PD	15.82 (7.91)	29.16 (3.51)	19.32 (7.68)	5.99 (4.24)	
	−2	NPD	15.54 (7.50)	28.27 (3.42)	19.29 (7.35)	5.53 (4.85)	.005
		PD	15.63 (7.89)	29.18 (3.51)	19.50 (7.60)	5.95 (4.35)	
Difference	0	NPD	5.29 (5.33)	6.74 (2.57)	5.46 (4.21)	4.05 (4.18)	<.001
		PD	5.75 (5.68)	7.49 (2.64)	5.70 (4.55)	4.95 (3.90)	
	−1	NPD	5.23 (5.30)	6.77 (2.56)	5.46 (4.15)	4.08 (4.21)	<.001
		PD	5.61 (5.68)	7.58 (2.76)	5.84 (4.58)	4.85 (3.88)	
	−2	NPD	5.14 (5.25)	6.78 (2.65)	5.48 (4.14)	4.15 (4.22)	<.001
		PD	5.55 (5.69)	7.61 (2.74)	5.94 (4.53)	4.76 (3.91)	

NPD: non-pneumothorax days, PD: pneumothorax days.

### 3.3. Comparisons of wind speed

The mean wind speeds (0.31 vs 0.48 m/s, 0.28 vs 0.46 m/s, and 0.20 vs 0.40 m/s), the minimum wind speeds (0.20 vs 0.31 m/s, 0.18 vs 0.25 m/s, and 0.16 vs 0.25 m/s), and the maximum wind speeds (0.44 vs 0.67 m/s, 0.36 vs 0.71 m/s, and 0.26 vs 0.58 m/s) were slower on Day 0, Day-1, and Day-2, respectively, in PD than in NPD during all 4 seasons. The change in wind speeds were smaller for all 3 days in PD than in NPD (0.23 vs 0.36 m/s, 0.19 vs 0.49 m/s, 0.10 vs 0.34 m/s) (Table 3).

**Table 3:** Comparison of wind speed between pneumothorax days and non-pneumothorax days adjusted by seasonal variations.

	Day	Group	Season				*P*
			Spring m/s (±SD)	Summer m/s (±SD)	Autumn m/s (±SD)	Winter m/s (±SD)	
Mean	0	NPD	2.62 (1.13)	2.40 (1.05)	2.21 (1.04)	2.58 (1.26)	<.001
		PD	2.32 (1.00)	2.07 (0.88)	1.88 (0.92)	2.10 (1.11)	
	−1	NPD	2.62 (1.13)	2.41 (1.04)	2.24 (1.06)	2.56 (1.23)	<.001
		PD	2.34 (1.12)	2.01 (0.82)	1.87 (0.92)	2.11 (1.17)	
	−2	NPD	2.60 (1.12)	2.41 (1.02)	2.23 (1.06)	2.54 (1.25)	<.001
		PD	2.40 (1.13)	2.02 (0.83)	1.89 (0.94)	2.14 (1.14)	
Min	0	NPD	0.83 (1.18)	0.83 (1.16)	0.69 (0.84)	0.91 (1.23)	<.001
		PD	0.64 (0.85)	0.61 (0.69)	0.49 (0.58)	0.60 (0.73)	
	−1	NPD	0.84 (1.19)	0.83 (1.13)	0.71 (0.89)	0.87 (1.11)	<.001
		PD	0.66 (0.92)	0.58 (0.66)	0.50 (0.59)	0.65 (0.99)	
	−2	NPD	0.83 (1.20)	0.83 (1.12)	0.71 (0.84)	0.87 (1.16)	<.001
		PD	0.67 (0.92)	0.58 (0.68)	0.50 (0.70)	0.63 (0.86)	
Max	0	NPD	4.94 (1.59)	4.40 (1.42)	4.15 (1.51)	4.74 (1.81)	<.001
		PD	4.46 (1.52)	3.96 (1.29)	3.71 (1.43)	4.07 (1.68)	
	−1	NPD	4.90 (1.56)	4.42 (1.43)	4.19 (1.53)	4.74 (1.76)	<.001
		PD	4.54 (1.59)	3.91 (1.23)	3.68 (1.42)	4.04 (1.75)	
	−2	NPD	4.88 (1.59)	4.42 (1.41)	4.17 (1.54)	4.69 (1.79)	<.001
		PD	4.62 (1.59)	3.91 (1.26)	3.73 (1.42)	4.11 (1.72)	
Difference	0	NPD	4.11 (1.44)	3.58 (1.26)	3.45 (1.29)	3.83 (1.54)	<.001
		PD	3.82 (1.34)	3.35 (1.11)	3.22 (1.24)	3.47 (1.39)	
	−1	NPD	4.07 (1.40)	3.59 (1.28)	3.48 (1.31)	3.88 (1.51)	<.001
		PD	3.88 (1.42)	3.33 (1.07)	3.19 (1.20)	3.39 (1.43)	
	−2	NPD	4.04 (1.43)	3.59 (1.27)	3.46 (1.31)	3.82 (1.50)	<.001
		PD	3.95 (1.41)	3.34 (1.07)	3.23 (1.21)	3.48 (1.45)	

NPD: non-pneumothorax days, PD: pneumothorax days.

### 3.4. Comparisons of atmospheric pressure

The mean atmospheric pressure (0.16 vs 0.52 hPa, 0.25 vs 0.42 hPa, and 0.34 vs 0.40 hPa) and the maximum atmospheric pressure (0.15 vs 0.53 hPa, 0.28 vs 0.49 hPa, and 0.33 vs 0.71 hPa) were greater on Day 0, Day-1, and Day-2, respectively, in PD than in NPD for all 4 seasons. The minimum atmospheric pressure was higher by 0.13 versus 0.43 hPa in PD than NPD. However, significant differences were observed for Day-1 (*P = *.023), while there were no significant differences in the minimum atmospheric pressures on Day 0 and Day-2. The change in atmospheric pressure was −0.04 versus 0.45 hPa greater in PD than in NPD; however, a significant difference was observed only on Day-2 (*P < *.001) (Table 4).

**Table 4:** Comparison of atmospheric pressure between pneumothorax days and non-pneumothorax days adjusted by seasonal variations.

	Day	Group	Season				*P*
			Spring hPa (±SD)	Summer hPa (±SD)	Autumn hPa (±SD)	Winter hPa (±SD)	
Mean	0	NPD	1007.60 (6.47)	999.54 (4.19)	1010.19 (5.87)	1015.71 (5.36)	.011
		PD	1007.99 (6.56)	1000.06 (4.32)	1010.35 (5.68)	1015.97 (4.97)	
	−1	NPD	1007.77 (6.52)	999.61 (4.09)	1010.00 (6.04)	1015.60 (5.33)	.009
		PD	1008.14 (6.54)	1000.03 (4.18)	1010.25 (5.90)	1015.92 (5.22)	
	−2	NPD	1007.92 (6.52)	999.65 (3.96)	1009.75 (6.12)	1015.58 (5.38)	.007
		PD	1008.32 (6.47)	999.99 (4.08)	1010.09 (6.07)	1015.92 (5.18)	
Min	0	NPD	1005.75 (5.94)	998.57 (4.54)	1008.21 (5.68)	1013.19 (5.69)	.062
		PD	1005.96 (6.09)	999.01 (4.62)	1008.36 (5.38)	1013.35 (5.41)	
	−1	NPD	1005.89 (6.03)	998.62 (4.41)	1008.00 (5.86)	1013.05 (5.72)	.023
		PD	1006.10 (6.00)	999.03 (4.48)	1008.32 (5.64)	1013.30 (5.56)	
	−2	NPD	1006.01 (6.03)	998.69 (4.32)	1007.83 (5.91)	1013.09 (5.73)	.077
		PD	1006.27 (5.91)	998.97 (4.30)	1008.08 (5.91)	1013.21 (5.58)	
Max	0	NPD	1009.47 (7.08)	1000.73 (3.77)	1012.23 (6.29)	1018.26 (5.41)	.009
		PD	1009.80 (7.27)	1001.26 (3.97)	1012.38 (6.10)	1018.66 (4.87)	
	−1	NPD	1009.62 (7.14)	1000.76 (3.72)	1012.01 (6.38)	1018.17 (5.34)	.004
		PD	1010.05 (7.24)	1001.25 (3.86)	1012.29 (6.22)	1018.56 (5.32)	
	−2	NPD	1009.69 (7.20)	1000.82 (3.62)	1011.77 (6.37)	1018.12 (5.44)	<.001
		PD	1010.41 (7.09)	1001.14 (3.82)	1012.13 (6.43)	1018.63 (5.15)	
Difference	0	NPD	3.72 (2.77)	2.16 (2.29)	4.02 (2.36)	5.07 (2.61)	.059
		PD	3.84 (2.79)	2.25 (2.42)	4.02 (2.24)	5.31 (2.69)	
	−1	NPD	3.73 (2.75)	2.14 (2.24)	4.01 (2.35)	5.12 (2.62)	.094
		PD	3.95 (2.82)	2.22 (2.43)	3.97 (2.15)	5.26 (2.78)	
	−2	NPD	3.68 (2.78)	2.13 (2.34)	3.94 (2.28)	5.03 (2.58)	<.001
		PD	4.14 (2.80)	2.17 (2.24)	4.05 (2.28)	5.42 (2.79)	

NPD: non-pneumothorax days, PD: pneumothorax days.

## 4. Discussion

Previous studies reported that spontaneous pneumothorax might occur in the form of group infection, which sociological factors cannot explain. Instead, it was assumed that weather conditions and the season might affect the incidence of SP.^[9,10]^ Based on this assumption, analyses of the differences in climate and environmental factors by comparing days with prevalent SP cases with days with few or no cases of SP were performed. In this study, we used data from large cities and assumed that the population densities of urban areas might affect the number of SP patients per city. Therefore, the days with more and less than 1 SP case per 1 million population were defined as PD and NPD, respectively. Before PD and NPD per season, the environmental factors were assessed for 3 days (Day 0, Day-1, and Day-2).

In Incheon, 465 (29.23%) patients with SP were observed during autumn. Meanwhile, in Busan, 480 (28.67%) patients were observed during spring, indicating a difference by region. Across the nation, the number of SP patients was the lowest during winter, with 4080 (22.86%) total patients. In one study, the number of SP patients was highest during autumn,^[6,11]^ while in another study, the highest number was observed in April.^[12]^ Conversely, Bertolaccini et al reported that the number of admitted pneumothorax patients was the lowest during winter.^[13]^ However, some studies reported no relationship between the incidence of SP and season.^[14,15]^

In this study, the maximum temperature and temperature changes were greater in PD than in NPD for all 3 days. Consistent with our findings, a previous study reported that the average temperature was higher by 2.43°C in PD than in NPD.^[11]^ In another study, a clear correlation between SP and temperature was observed when higher than 25°C.^[16]^ One reason may be that an increase in temperature leads to a decrease in atmospheric pressure. According to Boyle–Marriot’s law, this may result in air being trapped in the bullae, which may cause SP. In 1 univariate analysis study, the average, maximum, and minimum temperature was higher by 2.1°C, 2.4°C, and 1.8°C, respectively, in PD than in NPD.^[17]^ However, the multivariate logistic regression analysis performed in this study showed no association between SP and temperature, which agrees with other studies.^[18,19]^

The maximum, minimum, and average wind speeds and the change in wind speed were lower for all 3 days in PD than in NPD. In another study, the mean wind speed was 1.880 ± 0.8 m/s in PD, slower than 2.014 ± 0.6 m/s in NPD, by 0.134 m/s.^[6]^ This was explained by the difference in working and social lives on days with slower wind speeds for the evaluated regions. Moreover, a high wind speed was reported to directly affect the transpulmonary pressure gradient, leading to possible rupture of the blebs or bullae.^[13]^ However, other studies have reported that SP is not correlated with wind speed.^[17,20]^

In this study, the mean and maximum atmospheric pressures were greater in PD than in NPD, while the minimum and changes in atmospheric pressure were not consistently higher in PD for all 3 days. According to other studies, the correlation between high atmospheric pressure and SP can be explained using Boyle–Marriot’s law.^[13,21]^ In contrast, another study reported a correlation between low atmospheric pressure and SP,^[18]^ which demonstrated that the bullae in the lungs were affected by the check-valve system. Additionally, the association between SP and change in atmospheric pressure has been reported as well;^[16,17,20]^ but according to other studies, no relationship with SP was established.^[19]^

Several limitations must be considered when interpreting the findings in this study. First, this was conducted with a large target population in large areas over a long period of time. Thus, although the *P* value was significant, the differences in the mean values were not as great. Moreover, it was assumed that patients with SP with symptoms living in the same city were experiencing the same environmental conditions. Despite these limitations, this study is significant because it was conducted based on big national data.

In conclusion, high average and change in temperatures, slow and unchanging wind speeds, and high average and maximum atmospheric pressures for 3 days were associated with SP. Although many of our findings were consistent with previous studies, contradictory results have also been reported. These differences may be attributed to the different characteristics of the assessed population group, regional characteristics, and analysis methods used for each study. Therefore, studies demonstrating the correlation between environmental factors and SP are still lacking. However, the development of SP in clusters of patients, as well as the high or low frequency of SP during specific seasons, may suggest that SP is not only caused by a single environmental and climate factor but rather a complex interaction of multiple factors that can affect each patient in different manners. For future studies, multi-faceted research designs must be conducted to further assess the correlation between SP and environmental factors.

## Author contributions

**Conceptualization:** Suk Hee Lee, Young Woo Seo, Sang Gyu Kwak.

**Data curation:** Young Woo Seo, Sang Gyu Kwak.

**Formal analysis:** Young Woo Seo, Sang Gyu Kwak.

**Investigation:** Suk Hee Lee, Young Woo Seo.

**Methodology:** Suk Hee Lee, Young Woo Seo.

**Writing – original draft:** Suk Hee Lee, Young Woo Seo.

**Writing – review & editing:** Young Woo Seo.
